# Parthenolide attenuates LPS-induced activation of NF-κB in a time-dependent manner in rat myocardium^☆^

**DOI:** 10.1016/S1674-8301(12)60005-0

**Published:** 2012-01

**Authors:** Hong Xie, Chen Wang, Xuemei Wu, Xia Liu, Shigang Qiao, Chunfeng Liu, Hong Liu

**Affiliations:** a Department of Anesthesiology, the Second Affiliated Hospital of Soochow University, Suzhou 215004, China;; b Institute of Neuroscience, Soochow University, Suzhou 215004, China;; c Department of Anesthesiology and Pain Medicine, University of California Davis, Davis, CA 95616, USA.

**Keywords:** parthenolide, nuclear-factor-κB, lipopolysaccharide, myocardium

## Abstract

Parthenolide (PTN), a selective nuclear factor kappa B (NF-κB) inhibitor, has been used extensively to inhibit NF-κB activation. The duration of the inhibitory effect of PTN on NF-κB *in vivo* remains unclear. This study was to determine whether a lipopolysaccharide (LPS) challenge 6, 12 and 24 h after the administration of PTN could activate NF-κB. Rats were devided into five groups. The rats in the PTN, PTN+LPS and DMSO groups were injected intraperitoneally with PTN or DMSO. After 6, 12 or 24 h, LPS was administered in LPS and PTN+LPS groups. The expressions of NF-κB p50, IκBα and p-IκBα were inhibited in both PTN and PTN+LPS group at end of 6 and 12 h and no effects at 24 h. In summary, myocardial NF-κB expression occurs 1 h after the administration of LPS. PTN blocks this effect given at 6 h and no inhibitory effect 24 h after administration *in vivo*.

## INTRODUCTION

Nuclear factor-kappa B (NF-κB) participates in the regulation of multiple immediate early gene products involved in immune, acute phase and inflammatory responses, in addition to programmed cell death and cellular proliferation[1]. At present, NF-κB as a central event leading to the activation of sepsis and septic shock can be activated by a variety of pathogens known to cause septic shock syndrome[2].

Parthenolide (PTN), an NF-κB inhibitor, is responsible for many anti-inflammatory effects and has been used extensively in previous studies[3]-[8]. *In vitro* study has suggested that PTN can inhibit IKK and also directly inactivate NF-κB[4]. In addition, other studies have reported that PTN inhibits NF-κB activation in cultured cells and experimental models[5]-[8]. Evidence supports the concept that PTN can inhibit NF-κB activation by selective inhibition of IKK activation and IκBα degradation; however, data regarding effectiveness of PTN duration *in vivo* are currently unavailable.

Treating cells with lipopolysaccharide (LPS) results in the dissociation of cytoplasmic complexes containing NF-κB and the translocation of free NF-κB to the nucleus[9]. LPS is a primary inducer of inflammatory responses through the biosynthesis and release of a variety of inflammatory mediators via the mononuclear phagocyte system, which is involved in the immune inflammatory response, leading to sepsis and septic shock. NF-κB is a central mediator leading to sepsis and septic shock and can be activated by a variety of pathogens known to cause septic shock syndrome. The role of NF-κB activation in the pathophysiology of sepsis and the signal transduction pathways leading to NF-κB activation during sepsis has been intensively investigated[2]. NF-κB activity has been found to be markedly increased in every organ studied to date in both animal models of septic shock and humans with sepsis. The inhibition of NF-κB activation prevents multiple organ injury and improves survival in a rodent model of septic shock. Thus, NF-κB activation plays a central role in the pathophysiology of septic shock[2],[10]. PTN is a selective NF-κB inhibitor and protects against septic shock syndrome by selective inhibition of IKK activation and IκBα degradation[8]. In the present study, we investigated the specific role of NF-κB translocation in rat myocardium after an LPS challenge and determined whether the administration of PTN as a NF-κB inhibitor 6, 12 and 24 h prior could inhibit this effect.

## MATERIALS AND METHODS

### Grouping and experimental protocol

**Fig. 1:**
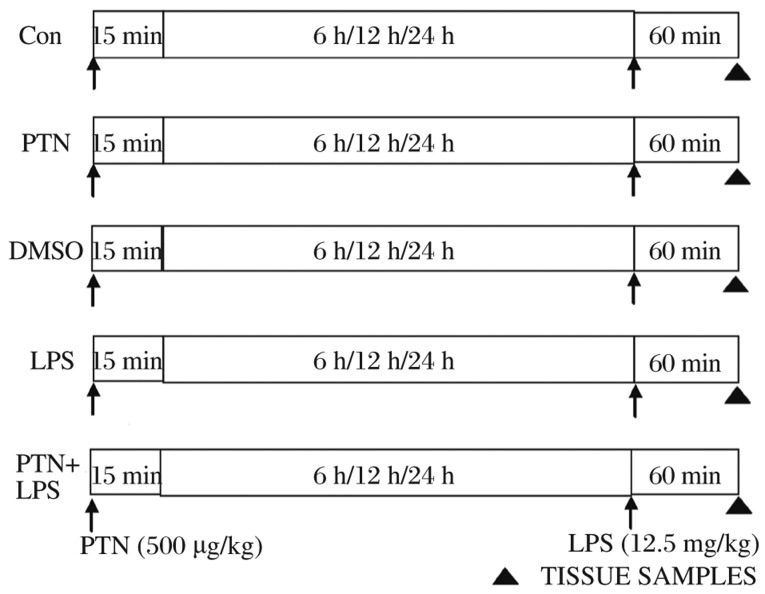
Schematic illustration of the experimental protocol used in Western immunoblotting experiments. Rats were divided into five groups at 6, 12 and 24 h after receiving intraperitoneally PTN, respectively: control CON, PTN, DMSO, LPS and PTN+LPS. LPS was administered subcutaneously at 6, 12 and 24 h after PTN injection. For Western immunoblotting measurements, the tissue samples were obtained 1 h after receiving LPS. PTN: parthenolide; DMSO: dimethylsulfoxide; LPS: lipopolysaccharide; NS: physiological normal saline.

This study was approved by the Animal Care Committee of the Medical College of Soochow University, and all experiments were conducted in accordance with the guidelines for animal care of the National Institutes of Health. A total of 90 adult male Sprague-Dawley rats (10-12 w, 250-350 g) were used in this study. ***Fig. 1*** illustrates the treatment groups in our study. We randomly devided the rats into 5 groups (*n* = 6 in each group) at 6, 12 and 24 h after receiving intraperitoneally (i.p.) PTN, respectively: control (CON), PTN, DMSO, LPS and PTN+LPS. The rats in the PTN, PTN+LPS and DMSO groups were injected i.p. with PTN (500 µg/kg) or DMSO as indicated, while the rats in the other groups were injected i.p. with an equivalent amount of saline[11]. At 6, 12 and 24 h after PTN injection, LPS (12.5 mg/kg) was administered subcutaneously to the LPS and PTN+LPS groups[12], while the rats in the other groups were injected subcutaneously with an equivalent volume of saline. Hearts were collected 1 h after LPS injection. The rats were anesthetized with an i.p. injection of sodium pentobarbital (50 mg/kg) and anticoagulated with an i.p. injection of heparin (1,000 U/kg). Left ventricular samples were quickly excised, cut into five or six cross-sectional slices of 2 mm thickness, snap frozen in liquid nitrogen and stored at -80°C for subsequent analysis.

### Western blotting analysis of NF-κB p50, IκBα and p-IκBα expression levels

Western immunoblotting was performed as described previously[13],[14]. Briefly, frozen tissue samples were homogenized using a polytron homogenizer (Beyotime Institute of Biotechnology, Haimen, China) in ice-cold lysis buffer with the complete protease inhibitor phenylmethylsulfonyl fluoride. After centrifugation at 15,000 *g* for 15 min at 4°C, total protein was isolated. The clarified supernatant was collected to quantify total protein expression. Protein concentrations were determined using an enhanced BCA protein assay kit (Beyotime Institute of Biotechnology). Equivalent amounts (100 µg) of protein samples were mixed with Laemmli buffer and heated at 100°C for 5 min, then separated by 5% to 10% sodium dodecyl sulfate polyacrylamide gel electrophoresis (SDS-PAGE) and transferred to nitrocellulose membranes (Pall Corporation, East Hills, USA) at 100 V for 1.5 h. The membranes were blocked with 5% nonfat dry milk in TBST (10 mmol/L Tris-HCl, pH 7.5, 150 mmol/L NaCl, 0.05% Tween-20) for 1 h at room temperature and immunoblotted overnight at 4°C with a rabbit polyclonal anti-NF-κB p50 antibody (Santa Cruz Biotechnology, Santa Cruz, USA), a rabbit polyclonal anti-phospho-IκBα antibody (Cell Signaling Co., Danvers, USA) or a mouse monoclonal anti-phospho-IκBα antibody (Santa Cruz Biotechnology) diluted 1:500 in 5% nonfat dry milk buffer. Membranes were then washed three times in TBST and incubated with a peroxidase-labeled antirabbit or anti-mouse IgG secondary antibody diluted 1:5,000 in 5% nonfat dry milk buffer (Cell Signaling Co.) for 1 h at room temperature. Membranes were washed three times in TBST before the antigen-antibody complexes were detected. Glyceraldehyde-3-phosphate dehydrogenase (GAPDH) was used as a reference for quantitative analysis. Specific antigen-antibody complexes were detected using an enhanced chemiluminescence system (ECL, Merck, Darmstadt, Germany). Protein densitometry was performed using Sigma Pro5.0 (Sigma, Saint Louis, USA).

### Statistical analysis

Data were shown as mean±SD. Statistical analyses were performed with SAS 8.1 software (SAS Institute Inc, Cary, USA). Differences between groups were evaluated using two-way analysis of variance, when appropriate, and the post hoc test used was the Newman-Keuls test. All *P* values were two-tailed and a *P* value < 0.05 was considered significant.

## RESULTS

### Expression of NF-κB p50 protein

**Fig. 2:**
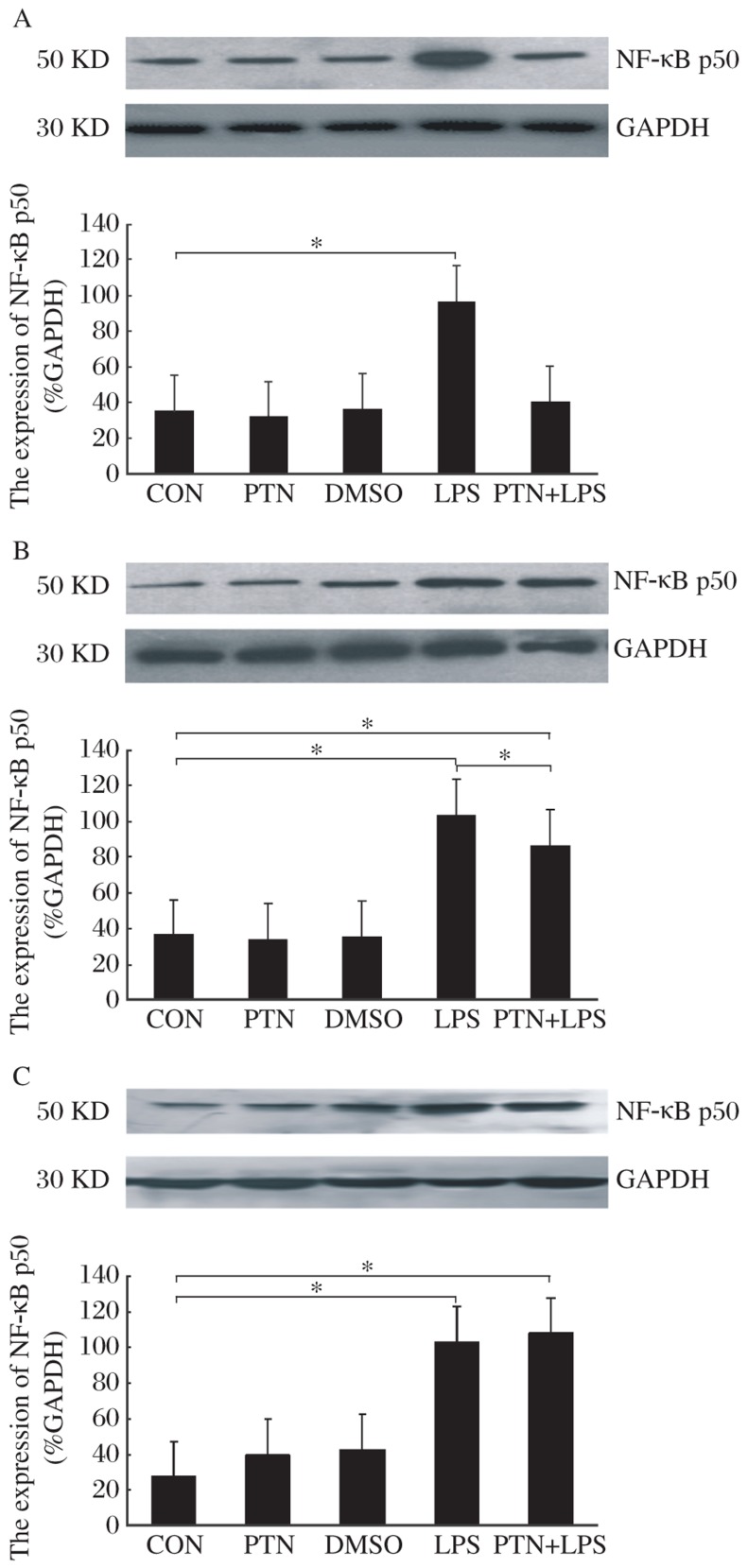
Representative effect of PTN on NF-κB p50 in the presence or absence of LPS. A, B and C stand for the expression of NF-κB p50 at 6, 12 and 24 h after receiving intraperitoneally PTN, respectively. Data are shown as the mean±SD, with *n* = 6 in each group. GAPDH: glyceraldehyde-3-phosphate dehydrogenase; PTN: parthenolide; DMSO: dimethylsulfoxide; LPS: lipopolysaccharide.

The NF-κB p50 protein at 6 h was up-regulated in the LPS group [(97±10)%, *P* < 0.05] in comparison with the CON group [(36±11)%], but there was no significant difference in the expression of NF-κB p50 in the PTN, DMSO and PTN+LPS groups [(32±9)%, (37±12)% and (41±13)%, respectively] compared to the CON group (***Fig. 2A***).

The NF-κB p50 protein at 12 h had no significant difference in the PTN and DMSO groups [(34±10)% and (36±19)%, respectively] compared to the CON group [(37±14)%], but the NF-κB p50 protein was up-regulated in the LPS and PTN+LPS groups [(104±4)% and (87±17)% respectively, *P* < 0.05] compared to the CON group. However, the up-regu-lated amplitude of the PTN+LPS group was smaller than that of the LPS group (*P* < 0.05, ***Fig. 2B***).

Western blot analysis showed that the NF-κB p50 protein at 24 h was up-regulated in the LPS and PTN+LPS groups [(103±17)% and (108±20)%, respectively, *P* < 0.05] 1 h after subcutaneous injection of LPS compared to the CON group [(27±10)%], but there was no significant difference in the expression of NF-κB p50 protein in the PTN and DMSO groups [(40±15)% and (43±14)%, respectively] compared to the CON group (***Fig. 2C***).

### Expression of IκBα protein

**Fig. 3:**
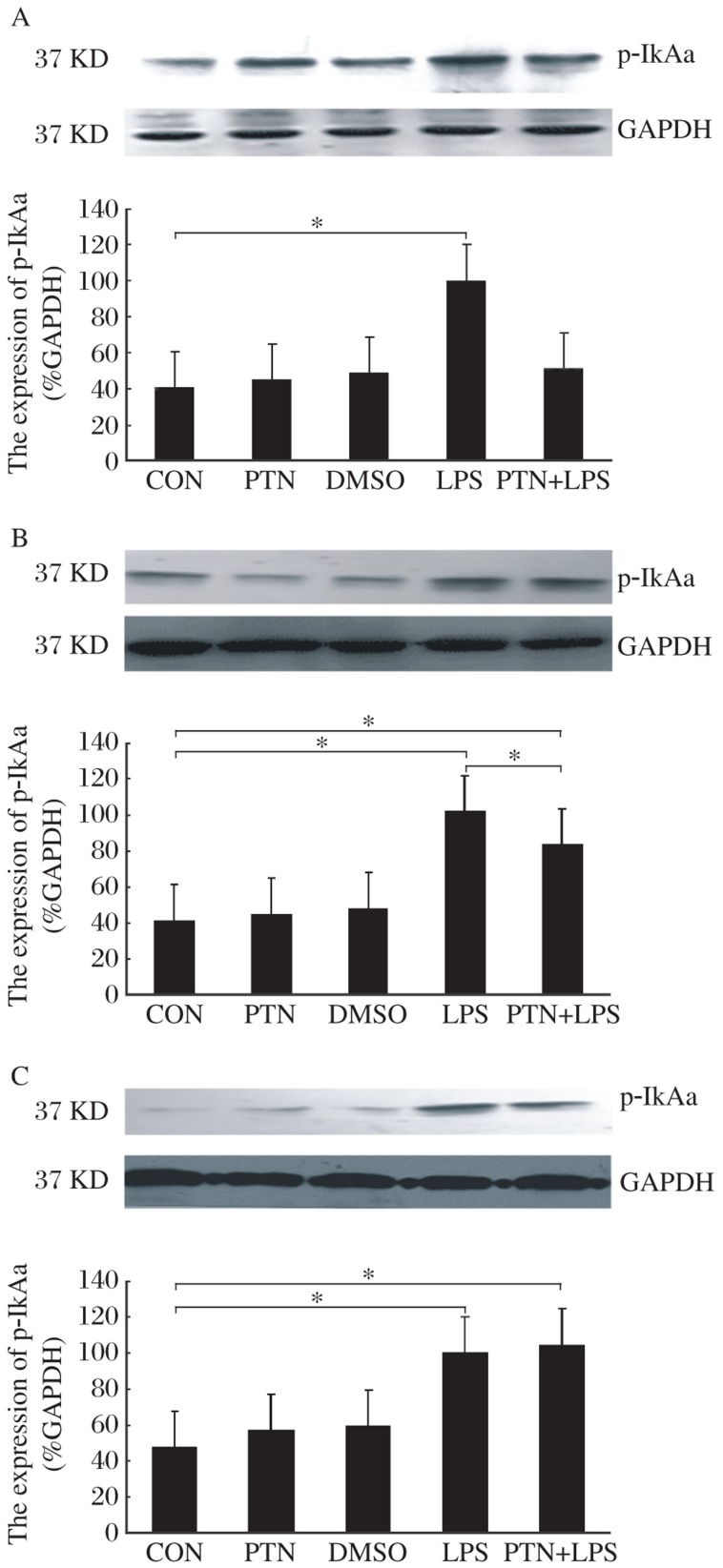
The effect of PTN on myocardial IκBα levels in the presence or absence of LPS. A, B and C stand for the expression of IκBα at 6, 12 and 24 h after receiving intraperitoneally PTN, respectively. Data are shown as the mean±SD, with *n* = 6 in each group. GAPDH: glyceraldehyde-3-phosphate dehydrogenase; PTN: parthenolide; DMSO: dimethylsulfoxide; LPS: lipopolysaccharide.

At 6 h, there were no changes in IκBα expression in the PTN, DMSO and PTN+LPS groups [(68±10)%, (71±12)%, and (65±14)% respectively] compared to the CON group [(69±9)%], but the IκBα protein expression of the LPS group [(39±7)%, *P* < 0.05] was down-regulated compared to the CON group (***Fig. 3A***).

At 12 h, the Western blotting analysis showed that the IκBα protein expression in the PTN and DMSO groups had no significant difference [(66±8)% and (63±10)%, respectively] compared to the CON group [(69±12)%], but in the LPS and PTN+LPS groups IκBα was decreased [(38±11)% and (52±7)%, respectively, *P* < 0.05] compared to the CON group. However, the amplitude in the PTN+LPS group was smaller than that of the LPS group (*P* < 0.05, ***Fig. 3B***).

At 24 h, Western blotting analysis revealed that there was no significant difference in IκBα expression in the PTN and DMSO groups [(85±9)% and (82±5)%, respectively, *P* > 0.05] compared to the CON group (80±11)% 1 h after injecting LPS subcutaneously. IκBα levels in the cytoplasmic fraction of the heart, however, decreased in the LPS and PTN+LPS groups [(38±5)% and (37±5)%, respectively, *P* < 0.05, ***Fig. 3C***].

### Expression of p-IκBα protein

**Fig. 4:**
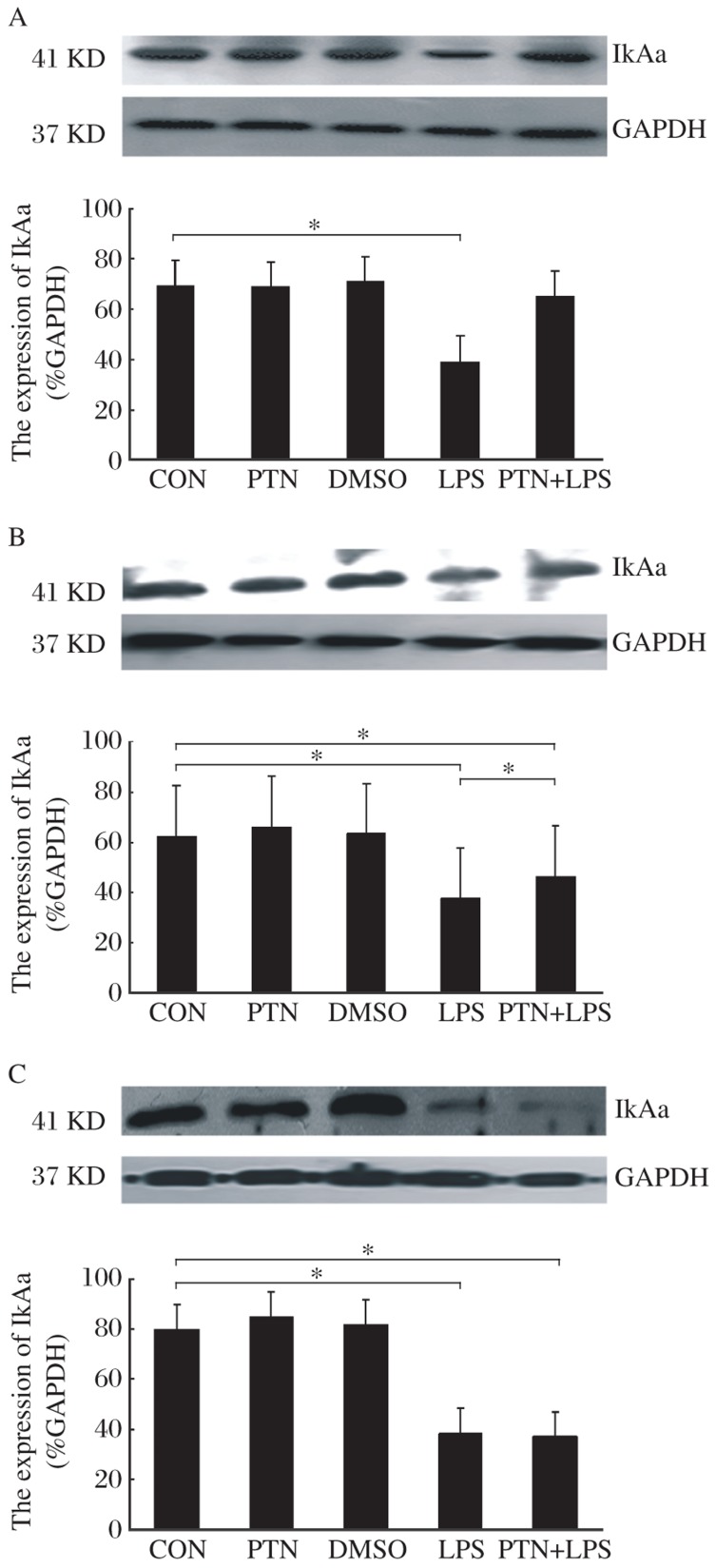
Representative effect of PTN on myocardial p-IκBα levels in the presence or absence of LPS. A, B and C stand for the expression of p-IκBα at 6, 12 and 24 h after receiving intraperitoneally PTN, respectively. Data are shown as the mean±SD, with *n* = 6 in each group. GAPDH: glyceraldehyde-3-phosphate dehydrogenase, PTN: parthenolide; DMSO: dimethylsulfoxide; LPS: lipopolysaccharide.

At 6 h, the Western blotting analysis showed that p-IκBα protein was up-regulated in the LPS group [(100±28)%, *P* < 0.05] compared to the CON group [(41±16)%], but there was no evident change of the expression of p-IκBα in the PTN, DMSO and PTN+LPS groups [(45±21)%, (49±24)% and (51±16)%, respectively] compared to the CON group (***Fig. 4A***).

At 12 h, the expression of p-IκBα had no significant difference in the CON, PTN and DMSO groups [(41±15)%, (45±19)% and (48±20)%, respectively], but the p-IκBα protein was up-regulated in the LPS and PTN+LPS groups [(102±16)% and (83±11)%, respectively, *P* < 0.05] compared to the CON group. However, the up-regulated amplitude in the p-IκBα protein of the PTN+LPS group was smaller than that of the LPS group (*P* < 0.05, ***Fig. 4B***).

At 24 h, p-IκBα expression was also determined by Western blot analysis. Compared to the CON group [(48±23)%], there was no significant difference in the expression of cytoplasmic p-IκBα in the PTN and DMSO groups [(57±23)% and (60±22)%, respectively]. Compared to the CON group [(48±23)%], however, the expression of p-IκBα was significantly increased in the LPS and PTN+LPS groups [(100±35)% and (105±36)%, respectively, *P* < 0.05, ***Fig. 4C***].

## DISCUSSION

It is well known that NF-κB transcription factors are critical regulators of inflammation, innate and adaptive immunity, and the suppression of apoptosis[15]. In resting cells, NF-κB is present in the cytosol as an inactive heterodimer, mainly consisting of the p65/p50 subunits bound to the IκB inhibitor, which is rapidly degraded in response to stimuli such as bacterial LPS, resulting in NF-κB nuclear entry[16]. Therefore, we assessed cardiac NF-κB activation after an LPS challenge and determined whether the administration of PTN, a selective NF-κB inhibitor, 6, 12 and 24 h prior to this challenge could inhibit the effects. Our results indicate that myocardial NF-κB can be activated following LPS administration, but PTN could block this effect completely when given 6 h and partly 12 h prior to the LPS challenge, and did not block NF-κB activation at 24 h. That means that PTN can block this activation when given within 24 h.

The activation of NF-κB is a key factor in excessive inflammatory responses and inflammatory injury. LPS is a primary inducer of inflammatory responses through the biosynthesis and release of a variety of inflammatory mediators, which is involved in the immune inflammatory response, leading to sepsis and septic shock[17]. Consistent with the theory that septic shock is a clinical syndrome with diverse etiologies, and NF-κB can be activated by a variety of bacteria, bacterial products and proinflammatory cytokines that are released during sepsis. NF-κB is the final target of these septic shock inducers[18].

NF-κB can be activated by numerous bacteria, bacterial toxins and proinflammatory mediators known to cause septic shock. LPS-induced activation of NF-κB is biphasic: an early phase occurs at 0.5-2 h post stimulation, and a late phase occurs at 8-12 h post stimulation. LPS and other early inflammatory mediators cause the early phase of NF-κB activation, while TNF and IL-1β mediate the late phase[19]. The effects of bacterial toxins on NF-κB activity are dramatic and widespread, leading to massive elevation in NF-κB activity in all organs studied to date[20]-[23]. This is consistent with the idea that multiple organs are involved in septic shock. In the heart, cardiac-specific overexpression of IκBα protects against cardiac injury during trauma[24], and the inhibition of NF-κB activation prevents LPS-induced increase in microvascular permeability[23]. Cultured cells or animals developing a tolerance to endotoxin exhibited down-regulated NF-κB activity and reduced expression of NF-κB-dependent genes when subsequently exposed to endotoxin[25]. This blunted NF-κB response is believed to be the result of increased IκBα[26], p50 or p52 expression or a switch from the formation of the p50/p65 heterodimer to the p50/p50 homodimer[27]. Studies using promoter deletion mutagenesis and reporter gene analysis have demonstrated that NF-κB plays a crucial role in the LPS-activated promoter activity of over 200 genes, many of which play important roles in the development of septic shock[28]-[30]. A major feature of the pathophysiology of septic shock is cardiovascular dysfunction; however, the inhibition of NF-κB activation corrects the cardiovascular functional abnormalities seen in septic shock. Studies have demonstrated that cardiac-specific overexpression of IκBα prevents LPS-induced repression of cardiac systolic and diastolic function[31]. Furthermore, inhibition of the NF-κB pathway can ameliorate the vascular derangement in both LPS and polymicrobial models of septic shock[32],[33].

The results of an endotoxemia study of jejunal mucosa induced by LPS suggest that activated NF-κB consists primarily of p50 subunits. As an inhibitor of NF-κB, IκB has a variety of forms, mainly consisting of IκBα and IκBβ. The main function of IκBα is to regulate and control the activation of NF-κB. IκBα demonstrates rapid degradation and resynthesis in response to stimuli such as LPS and TNF-α and is responsible for the acute-phase activation of NF-κB. In contrast, IκBβ demonstrates delayed degradation and resynthesis and is responsible for the persistence of NF-κB activation[12]. This process requires the phosphorylation of IκB by the IKK complex and the activation of NF-κB in response to proinflammatory stimuli and pathogen-associated molecular patterns[15],[16]. In this study, therefore, we determined whether the model of septic shock was successful by measuring the changes of NF-κB p50 and IκBα expression 1 h after the administration of LPS. Our results show that the majority of NF-κB p50 is activated 1 h after the administration of LPS, inducing corresponding changes in the expression of IκBα and p-IκBα.

PTN has attracted considerable interest, and PTN-containing products are commonly used to treat a variety of inflammatory conditions such as migraine, rheumatoid arthritis and asthma[34]. Several studies have reported that PTN inhibits NF-κB activation in cultured cells and experimental models[5]-[8],[35]. Furthermore, in vitro experiments with LPS-stimulated vascular smooth muscle cells and monocytes have shown that PTN at noncytotoxic doses inhibits IκBα degradation, preventing NF-κB activation and the subsequent gene expression. PTN also has beneficial effects during myocardial ischemia, endotoxic shock and renal disease through NF-κB inhibition[8]. Studies have found that PTN prevents NF-κB activation and dependent gene expression in both human and murine cells[36], and evidence indicates that PTN may inhibit the phosphorylation and degradation of IκBα[8], directly affect the DNA-binding ability of NF-κB without changing IκBα degradation or block a signaling pathway other than NF-κB. Both the in vitro and in vivo anti-inflammatory effects of PTN have been associated with the inhibition of IκBα depletion, which in turn results in the inhibition of excessive activation of NF-κB[37]. Some studies have reported that PTN protects against myocardial ischemia and reperfusion injury in rats by selective inhibition of IKK activation and IκBα degradation[8]. In this study, the expression levels of NF-κB, IκBα and p-IκBα in the LPS group at 6 h were different from those in the PTN+LPS group, which means that PTN blocked the activation of NF-κB induced by LPS within 6 h. The expression levels of NF-κB, IκBα and p-IκBα in the LPS and PTN+LPS groups had a similar trend at 12 h, but amplitude was different between them, showing that PTN has a partial effect in blocking NF-κB activation. Inversely, the expression levels of NF-κB, IκBα and p-IκBα in the PTN+LPS group were similar to those in the LPS group and different from those in the PTN group when given at 24 h prior to LPS administration, which means that PTN did not block the activation of NF-κB induced by LPS at 24 h. From what has been discussed above, it would be reasonable to believe that PTN blocked the activation of NF-κB induced by LPS within 6 and 12 h, not at 24 h.

Our study has several potential limitations. We did not specifically investigate different doses of PTN in the pharmacological inhibition of NF-κB (500 µg/kg) for the second window of protection (SWOP)[8]. In addition, we focused on a single organ, the heart, at only a 1-h interval of LPS challenge. In future studies, we will design and execute a comprehensive approach to accomplish our original goal of determining the duration of PTN following *in vivo* administration.

In conclusion, myocardial NF-κB expression occurs 1 h after the administration of LPS, and PTN, a selective inhibitor of NF-κB, could block this effect completely when given at 6 h, and partly block the activity 12 h prior to the LPS challenge. There was no block after 24 h. This further indicates that PTN has no inhibitory effect 24 h after administration.

## References

[1] Fan C, Li Q, Zhang Y, Liu X, Luo M, Abbott D (2004). IkappaBalpha and IkappaBbeta possess injury context-specific functions that uniquely influence hepatic NF-kappaB induction and inflammation. J Clin Invest.

[2] Liu SF, Malik AB (2006). NF-κB activation as a pathological mechanism of septic shock and inflammation. Am J Physiol Lung Cell Mol Physiol.

[3] Jain NK, Kulkarni SK (1999). Antinociceptive and anti-inflammatory effects of Tanacetum parthenium L. extract in mice and rats. J Ethnopharmacol.

[4] Kwok BH, Koh B, Ndubuisi MI, Elofsson M, Crews CM (2001). The anti-inflammatory natural product parthenolide from the medicinal herb Feverfew directly binds to and inhibits IkappaB kinase. Chem Biol.

[5] Hehner SP, Heinrich M, Bork PM, Vogt M, Ratter F, Lehmann V (1998). Sesquiterpene lactones specifically inhibit activation of NF-kappa B by preventing the degradation of I kappa B-alpha and I kappa B-beta. J Biol Chem.

[6] Martin-Ventura JL, Ortego M, Esbrit P, Hernández-Presa MA, Ortega L, Egido J (2003). Possible role of parathyroid hormone-related protein as a proinflammatory cytokine in atherosclerosis. Stroke.

[7] Sheehan M, Wong HR, Hake PW, Malhotra V, O'Connor M, Zingarelli B (2002). Parthenolide, an inhibitor of the nuclear factor-kappaB pathway, ameliorates cardiovascular derangement and outcome in endotoxic shock in rodents. Mol Pharmacol.

[8] Zingarelli B, Hake PW, Denenberg A, Wong HR (2002). Sesquiterpene lactone parthenolide, an inhibitor of IkappaB kinase complex and nuclear factor-kappaB, exerts beneficial effects in myocardial reperfusion injury. Shock.

[9] Paparella D, Yau TM, Young E (2002). Cardiopulmonary bypass induced inflammation: pathophysiology and treatment. An update. Eur J Cardiothorac Surg.

[10] Ye X, Ding J, Zhou X, Chen G, Lin SF (2008). Divergent roles of endothelial NF-kappaB in multiple organ injury and bacterial clearance in mouse models of sepsis. J Exp Med.

[11] Wang C, Xie H, Liu X, Qin Q, Wu X, Liu H (2010). Role of nuclear factor-kappaB in volatile anaesthetic preconditioning with sevoflurane during myocardial ischaemia/reperfusion. Eur J Anaesthesiol.

[12] Pritts TM, Moon R, Fischer JE, Salzmam AL, Hasselgren PO (1998). Nuclear factor-kB is activated in intestinal mucoma during endotoxemia. Arch Surg.

[13] Chiari PC, Bienengraeber MW, Pagel PS, Krolikowski JG, Kersten JR, Warltier DC (2005). Isoflurane protects against myocardial infarction during early reperfusion by activation of phosphatidylinositol-3-kinase signal transduction: evidence for anesthetic-induced postconditioning in rabbits. Anesthesiology.

[14] Xie Y, Yang H, Miller JH, Shih DM, Hicks GG, Xie J (2008). Cells deficient in oxidative DNA damage repair genes Myh and Ogg1 are sensitive to oxidants with increased G2/M arrest and multinucleation. Carcinogenesis.

[15] Karin M, Lin A (2002). NF-kappaB at the crossroads of life and death. Nat Immunol.

[16] Ghosh S, Karin M (2002). Missing pieces in the NF-kappaB puzzle. Cell.

[17] Anrather J, Racchumi G, Iadecola C (2005). Cis-acting, element-specific transcriptional activity of differentially phosphorylated nuclear factor-kappa B. J Biol Chem.

[18] Ulevitch RJ (2004). Therapeutics targeting the innate immune system. Nat Rev Immunol.

[19] Han SJ, Ko HM, Choi JH, Seo KH, Lee HS, Choi EK (2002). Molecular mechanisms for lipopolysaccharide-induced biphasic activation of nuclear factor-kappa B (NF-kappa B). J Biol Chem.

[20] Kelly A, Vereker E, Nolan Y, Brady M, Barry C, Loscher CE (2003). Activation of p38 plays a pivotal role in the inhibitory effect of lipopolysaccharide and interleukin-1 beta on long term potentiation in rat dentate gyrus. J Biol Chem.

[21] Liu SF, Ye X, Malik AB (1999). Pyrrolidine dithiocarbamate prevents I-kappaB degradation and reduces microvascular injury induced by lipopolysaccharide in multiple organs. Mol Pharmacol.

[22] Pocock J, Gomez-Guerrero C, Harendza S, Ayoub M, Hernández-Vargas P, Zahner G (2003). Differential activation of NF-kappa B, AP-1, and C/EBP in endotoxin-tolerant rats: mechanisms for in vivo regulation of glomerular RANTES/CCL5 expression. J Immunol.

[23] Pritts TA, Moon MR, Wang Q, Hungness ES, Salzman AL, Fischer JE (2000). Activation of NF-kappaB varies in different regions of the gastrointestinal tract during endotoxemia. Shock.

[24] Carlson DL, White DJ, Maass DL, Nguyen RC, Giroir B, Horton JW (2003). I kappa B overexpression in cardiomyocytes prevents NF-kappa B translocation and provides cardioprotection in trauma. Am J Physiol Heart Circ Physiol.

[25] Medvedev AE, Kopydlowski KM, Vogel SN (2000). Inhibition of lipopolysaccharide-induced signal transduction in endotoxin-tolerized mouse macrophages: dysregulation of cytokine, chemokine, and toll-like receptor 2 and 4 gene expression. J Immunol.

[26] Shames BD, Meldrum DR, Selzman CH, Pulido EJ, Cain BS, Banerjee A (1998). Increased levels of myocardial IkappaB-alpha protein promote tolerance to endotoxin. Am J Physiol.

[27] Bohuslav J, Kravchenko VV, Parry GC (1998). Regulation of an essential innate immune response by the p50 subunit of NF-kappaB. J Clin Invest.

[28] Baeuerle PA, Baichwal VR (1997). NF-kappa B as a frequent target for immunosuppressive and anti-inflammatory molecules. Adv Immunol.

[29] Brown MA, Jones WK (2004). NF-kappaB action in sepsis: the innate immune system and the heart. Front Biosci.

[30] Pahl HL (1999). Activators and target genes of Rel/NF-kappaB transcription factors. Oncogene.

[31] Haudek SB, Spencer E, Bryant DD, White DJ, Maass D, Horton JW (2001). Overexpression of cardiac I-kappaBalpha prevents endotoxin-induced myocardial dysfunction. Am J Physiol Heart Circ Physiol.

[32] Liu SF, Ye X, Malik AB (1997). *In vivo* inhibition of nuclear factor-kappa B activation prevents inducible nitric oxide synthase expression and systemic hypotension in a rat model of septic shock. J Immunol.

[33] Sheehan M, Wong HR, Hake PW, Zingarelli B (2003). Parthenolide improves systemic hemodynamics and decreases tissue leukosequestration in rats with polymicrobial sepsis. Crit Care Med.

[34] Pajak B, Gajkowska B, Orzechowski A (2008). Molecular basis of parthenolide-dependent proapoptotic activity in cancer cells. Folia Histochem Cytobiol.

[35] Konia MR, Schaefer S, Liu H (2009). Nuclear factor-[kappa]B inhibition provides additional protection against ischaemia/reperfusion injury in delayed sevoflurane preconditioning. Eur J Anaesthesiol.

[36] Tanaka K, Hasegawa J, Asamitsu K, Okamoto T (2005). Prevention of the ultraviolet B-mediated skin photoaging by a nuclear factor kappaB inhibitor, parthenolide. J Pharmacol Exp Ther.

[37] Saadane A, Masters S, DiDonato J, Li J, Berger M (2007). Parthenolide inhibits IkappaB kinase, NF-kappaB activation, and inflammatory response in cystic fibrosis cells and mice. Am J Respir Cell Mol Biol.

